# A Potential New Pathway for PD-L1 Costimulation of the CD8-T Cell Response to *Listeria monocytogenes* Infection

**DOI:** 10.1371/journal.pone.0056539

**Published:** 2013-02-11

**Authors:** Daqi Xu, Han-Hsuan Fu, Joshua J. Obar, Jang-June Park, Koji Tamada, Hideo Yagita, Leo Lefrançois

**Affiliations:** 1 Center for Integrated Immunology and Vaccine Research, Department of Immunology, University of Connecticut Health Center, Farmington, Connecticut, United States of America; 2 Marlene and Stewart Greenebaum Cancer Center, University of Maryland, Baltimore, Maryland, United States of America; 3 Department of Immunology, Juntendo University School of Medicine, Tokyo, Japan; 4 Department of Immunology and Infectious Diseases, Montana State University, Bozeman, Montana, United States of America; Leiden University Medical Center, The Netherlands

## Abstract

Programmed death ligand-1 (PD-L1) is an important negative regulator of T cell immune responses via interactions with PD-1 and CD80. However, PD-L1 can also act as a positive costimulator, but the relevant counterreceptor is not known. We analyzed the role of PD-L1 in CD8-T cell responses to infection with *Listeria monocytogenes (LM)* or vesicular stomatitis virus (VSV). PD-L1 blockade impaired antigen-specific CD8 effector T cell expansion in response to LM, but not to VSV infection, particularly limiting short-lived effector cell differentiation. Simultaneous CD4-T cell depletion and anti-PD-L1 blockade revealed that PD-L1 provided costimulation even in the absence of CD4-T cells. Most importantly, specific blockade of PD-L1 binding to CD80 or to PD-1 did not recapitulate PDL-1 blockade. The results suggested that PD-L1 plays an important costimulatory role for antigen-specific CD8 T cells during *LM* infection perhaps through a distinct receptor or interaction epitope.

## Introduction

Optimal T cell activation requires three signals: 1) interaction between TCR and the cognate peptide-MHC complex, 2) positive costimulation of antigen-specific T cells to promote expansion and survival [1]; and 3) cytokines that facilitate T cell differentiation, expansion, and survival [2]. Besides positive costimulation, there are coinhibitory signals crucial for maintaining immune system homeostasis and limiting deleterious inflammatory responses as well as autoimmunity [3]. The B7:CD28 costimulatory family consists of both positive and negative costimulatory molecules including CD28, CTLA4 and their ligands CD80 (B7.1) and CD86 (B7.2), and programmed death-1 (PD-1) and its ligands PD-L1 and PD-L2. Programmed death-1 (PD-1) binds to both PD-L1 and PD-L2 and is upregulated after T cell activation which serves to minimize inflammatory side-effects[4]. PD-1 also acts to limit immunity during chronic virus infection such that blocking PD-1 or PD-L1 can result in reversal of T cell exhaustion and viral clearance [5], [6]. In a T cell tolerance model, blocking PD-L1 augmented T cell expansion and function as compared to PD-1 blockade[7]. This difference implied the possible existence of a second receptor for PD-L1, which was subsequently identified as CD80 [5], [8]. In addition, it was recently demonstrated that the PD-L1:CD80 interaction promotes peripheral tolerance [7].

In contrast to the inhibitory roles played by the PD-1 pathway, PD-L1 can also serve as a positive costimulator. PD-L1 interactions promote bacterial clearance [9]–[11], Th1 differentiation and expansion[12] and the development of colitis [13]. In the current study, we investigated the role of PD-L1 in the regulation of the endogenous antigen-specific CD8 and CD4 T cell responses to bacteria and virus infections. We unveiled a costimulatory role for PD-L1 in the CD8 T cell response to *Listeria monocytogenes* (LM), but not to vesicular stomatitis virus (VSV) infection. PD-L1 signaling augmented the proliferation of responding CD8 T cells and modulated differentiation of the short-lived effector cell subset via a CD4 T cell independent mechanism. Moreover, PD-L1 signals appeared to be delivered through a PD-1 and CD80 independent pathway, thereby suggesting the possible existence of an additional PD-L1 ligand.

## Methods

### Mice and infections

C57BL/6 mice were purchased from the National Cancer Institute. All animal protocols were approved by the University of Connecticut Health Center Animal Care Committee. Mice were infected with 1×10^3^ cfu LM-OVA or 1×10^5^ pfu of VSV-ova i.v.

### mAb treatment

Mice were treated with 200 µg mAb specific for PD-L1 (10F.9G2 [14]), PD-L2 (TY25 [15]), PD-1 (RMP1-14 [16]) or 43H12 (PD-L1-CD80 [7]), i.p. on day -1 and every other day after infection. CD4 T cell depletion was done by treating mice with 200 µg GK1.5 i.p. 3 days before infection and every other day after infection.

### BrdU incorporation assay

Mice were treated with 1 mg BrdU i.p. 16 hr before sacrificing. Staining of BrdU incorporation followed the BrdU Flow kit protocol (Becton-Dickinson).

### Flow cytometry

Single-cell suspensions were prepared by collagenase digestion as previously described [17]. Lymphocytes (5×10^6^ cells/ml) were stained with peptide:MHC tetramers, and other antibodies as indicated. The LLO-I-A^b^ tetramer [18] was generously provided by Dr. Marc Jenkins (UMINN).

### Statistical analysis

Statistical significance was determined with unpaired *t*-test. For bacterial counts statistical significance was determined with a Mann-Whitney test, and for data containing more than 2 groups, a one-way ANOVA test was applied by GraphPad Prism.

## Results and Discussion

### Upregulation of PD-L1 on CD8 T cells after primary LM or VSV infection

**Figure 1:**
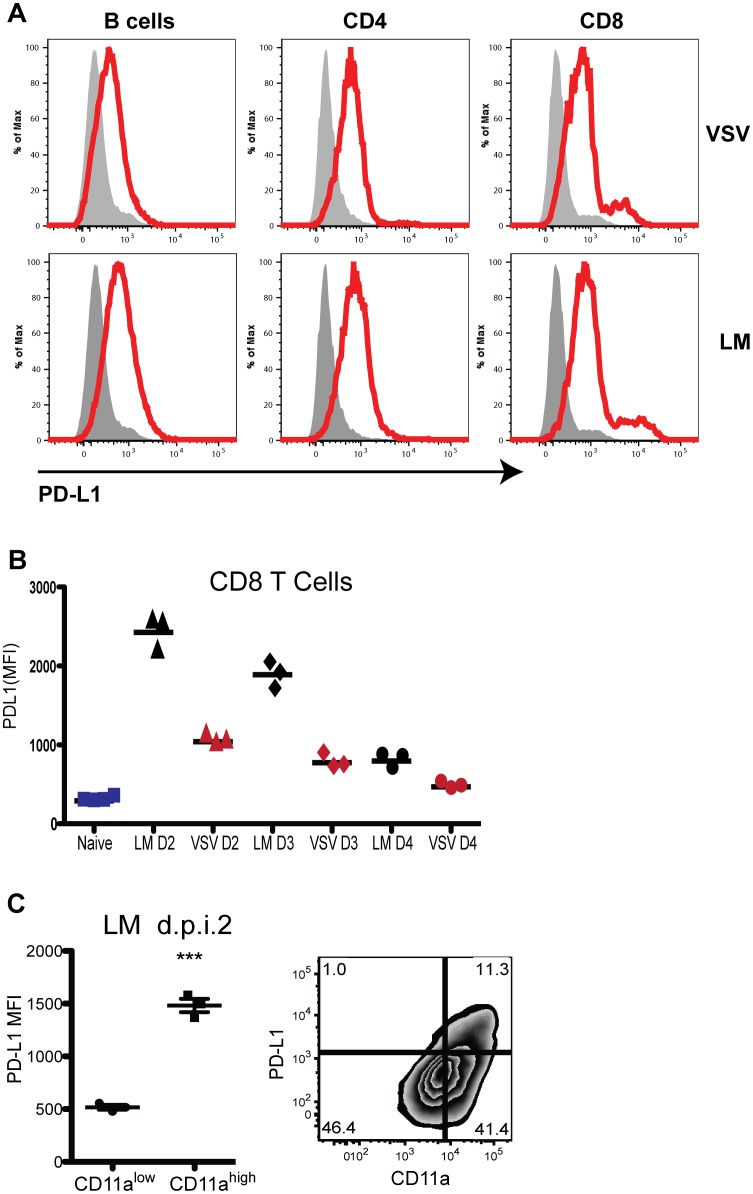
PD-L1 induction in response to infection. A, PD-L1 expression on CD4 T, CD8 T, and B cells on day 2 after LM or VSV infection. Filled histogram: naive control. Open histogram: day 2 after LM or VSV i.v. infection. B, Comparison of PD-L1 expression on total CD8 T cells 2 days after LM or VSV infection. C, Comparison of PD-L1 expression by naïve (CD11a^low^) and activated/memory (CD11a^high^) CD8 T cells and representative 2-D plot of CD11a versus PD-L1 expression. Data were analyzed by Student's *t* test. (***p<0.001). Gating strategy for T cells is based on CD4, CD8 and CD3 expression. Data are representative of three independent experiments with five mice per group.

We examined PD-L1 expression after i.v. infection with LM-ova or VSV-ova. Two days after infection with either pathogen, PD-L1 was markedly upregulated on bulk CD4 T cells, CD8 T cells, and B cells (Fig.1A). PD-L1 expression on CD8 T cells peaked ∼day 2 post-infection and subsequently declined (Fig. 1B). *LM* infection induced higher levels of PD-L1 on bulk CD8 T cells as compared to levels induced by VSV infection (Fig. 1B). Moreover, CD11a^high^ effector/memory phenotype CD8 T cells expressed substantially more PD-L1 as compared to their CD11a^low^ naïve counterparts (Fig. 1C). Indeed, high PD-L1 expression correlated with high CD11a levels (Fig. 1C). Thus, PD-L1 expression was transiently upregulated on T cells after LM infection, similar to other costimulatory molecules [19], [20].

### PD-L1 blockade inhibits the CD8 T cell response to LM infection

**Figure 2:**
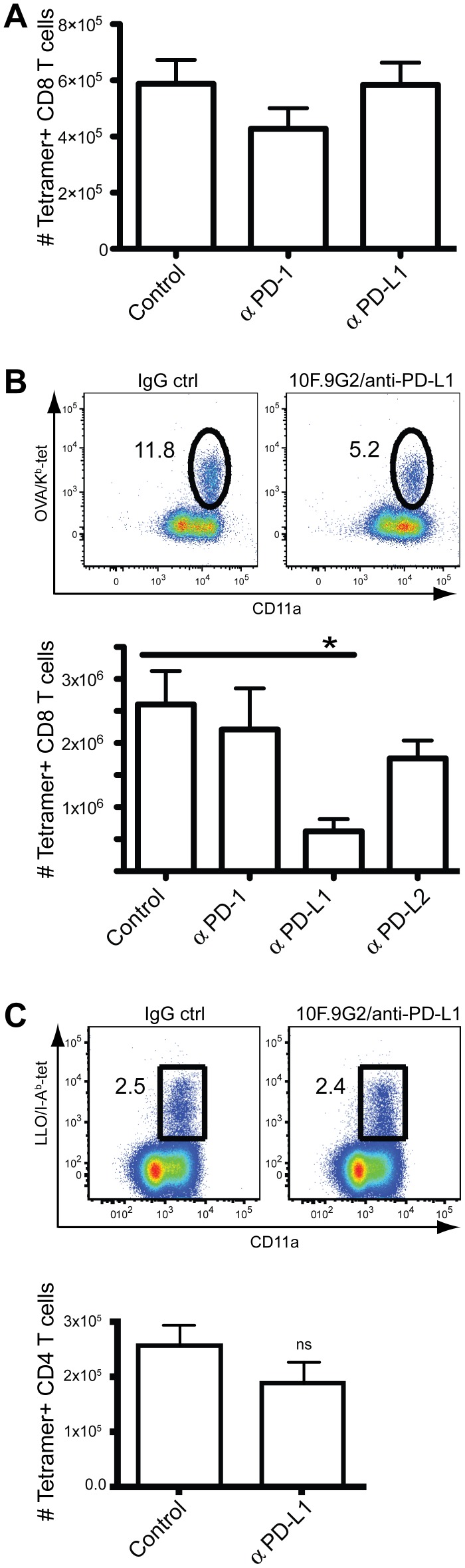
PD-L1 costimulates the CD8 T cell response to LM infection. A, OVA_257–264_/K^b+^ splenic CD8 T cell population seven days after VSV-OVA infection from mice treated with IgG isotype control, anti-PD-1 (RMP1-14), or anti-PD-L1 (10F.9G2). B, (Top panel) Representative dot-plot of the CD8 T cell response from control or anti-PD-L1 treated mice eight days after infection. (Bottom panel) Compiled data showing the total numbers of OVA_257–264_/K^b+^ splenic CD8 T cells eight days after LM-OVA infection from mice treated with IgG isotype control, anti-PD-1(RMP1-14), anti-PD-L1(10F.9G2), or anti-PD-L2 (TY25). Data were analyzed by two-way ANOVA. (*p<0.05. ns, non significant). C, (Top panel) Representative dot-plot of the splenic CD4 T cell response from control or anti-PD-L1 treated mice eight days after infection. (Bottom panel) Compiled data showing the total numbers of LLO_190–201_/I–A^b+^ CD4 T cells of the spleen from day 8 LM infected mice treated with anti-PD-L1 (10F.9G2) compared with IgG isotype control. Data were analyzed by Student's *t* test. Data are representative of three independent experiments with five mice per group.

**Figure 3:**
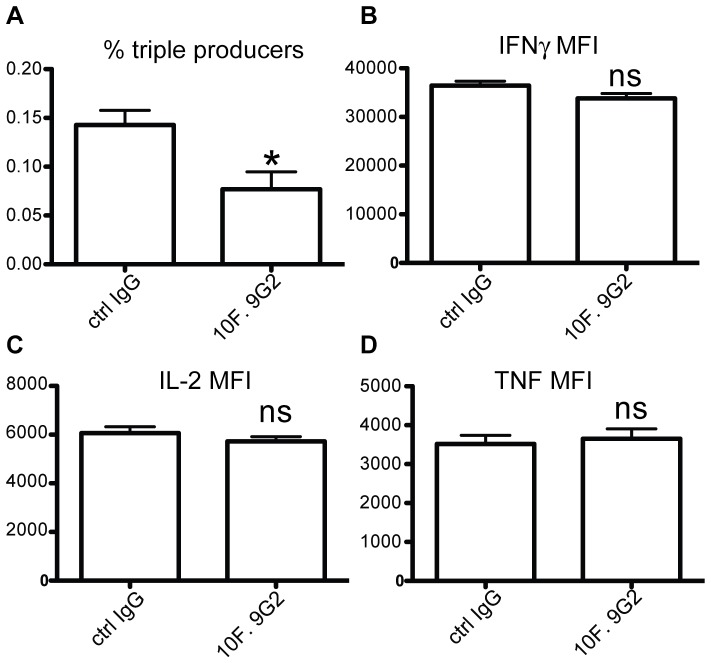
PD-L1 enhances multifunctional effector CD8 T cell generation. Mice were infected i.v. with 1000 cfu LM-OVA and treated with anti-PD-L1 or control IgG. Eight days later splenocytes were stimulated *in vitro* with SIINFEKL peptide for 5 hours in the presence of brefeldin A. Production of IL-2, IFNγ and TNF was measured by intracellular staining and flow cytometry. A. The frequency of IFNγ^+^TNF^+^IL-2^+^ antigen-specific CD8^+^ T cells. B–D. Comparison of the mean fluorescent intensity (MFI) of staining for each cytokine. Values are means +/− standard error. Data are representative of three independent experiments with five mice per group. Data were analyzed by student *t* test. (*p<0.05, ns, not significant).

To test the potential role of the PD-1 axis in the antigen-specific CD8 T cell response, we treated mice with anti-PD-L1 (10F.9G2), anti-PD-L2 (TY25), or anti-PD-1 (RMP1-14) blocking mAb throughout the infection. The pMHCI tetramer-OVA_257–264_/K^b^ was used to identify antigen-specific CD8 T cells on day 8 post LM-ova or day 7 post-VSV-ova infections, near the peak of the responses. The VSV-specific CD8 T cell response was not affected by either anti-PD-L1, –PD-L2, or -PD-1 mAbs (Fig. 2A and data not shown). In contrast, blocking PD-L1 resulted in an ∼80% inhibition of the anti-LM CD8 T cell response, while PD-L2 or PD-1 blockade had no effect (Fig. 2B). Interestingly, the LLO_190–201_/I-A^b^-specific CD4-T cell response was not diminished by PD-L1 blockade (Fig. 2C), indicating that a loss of CD4 T cell help could not explain the inhibition of the CD8 T cell response. We also examined the production of cytokines after PD-L1 blockade. While the overall number of cytokine producing cells decreased after PD-L1 blockade, as expected based on the loss of tetramer+ cells, the cells that produced IFNγ, TNF, or IL-2 did so at levels comparable to their normal counterparts (Fig. 3B–D). However, the percentage of polyfunctional antigen-specific CD8 T cells, i.e. those that produced all three cytokines, was reduced by PD-L1 blockade (Fig. 3A). Thus, PD-L1 controlled both the magnitude and the functionality of the CD8 T cell response to LM infection.

**Figure 4:**
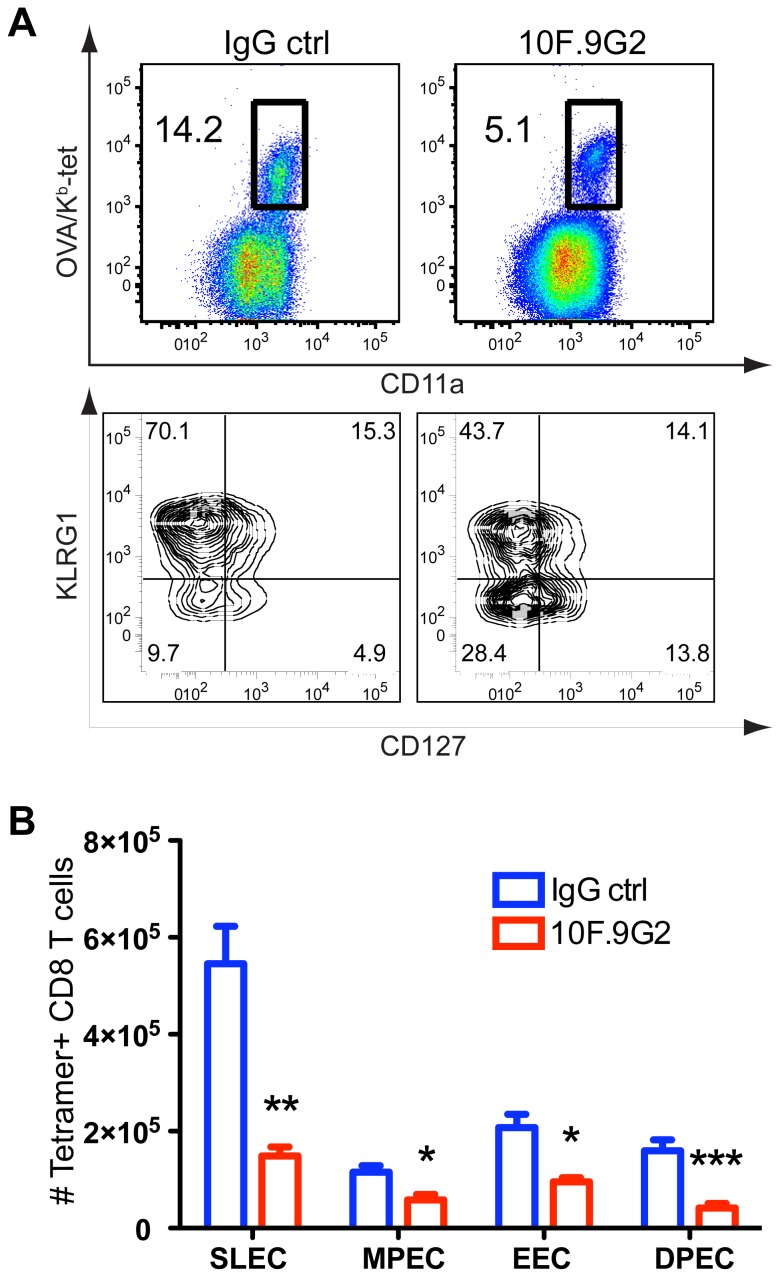
PD-L1 costimulation facilitates SLEC differentiation. Eight days after LM-OVA infection, SLEC, MPEC, DPEC and EEC population was analyzed within OVA_257–264_/K^b+^ splenic CD8 T cell population according to their KLRG1 and IL-7R expression. A, representative plots of the OVA-specific CD8 T cell response and the expression of CD127 and KLRG1 by gated tetramer+ cells with or without PD-L1 blockade. B, Graphs show the compiled proportion of each subset with or without anti-PD-L1 blockade (SLEC: KLRG1+ IL-7R-; MPEC: KLRG1−, IL−7R+; EEC: KLRG1−, IL−7R−; DPEC: KLRG1+, IL−7R+). Data are representative of three independent experiments with five mice per group. (*p<0.05, **p<0.01, ***p<0.001, ns, not significant).

**Figure 5:**
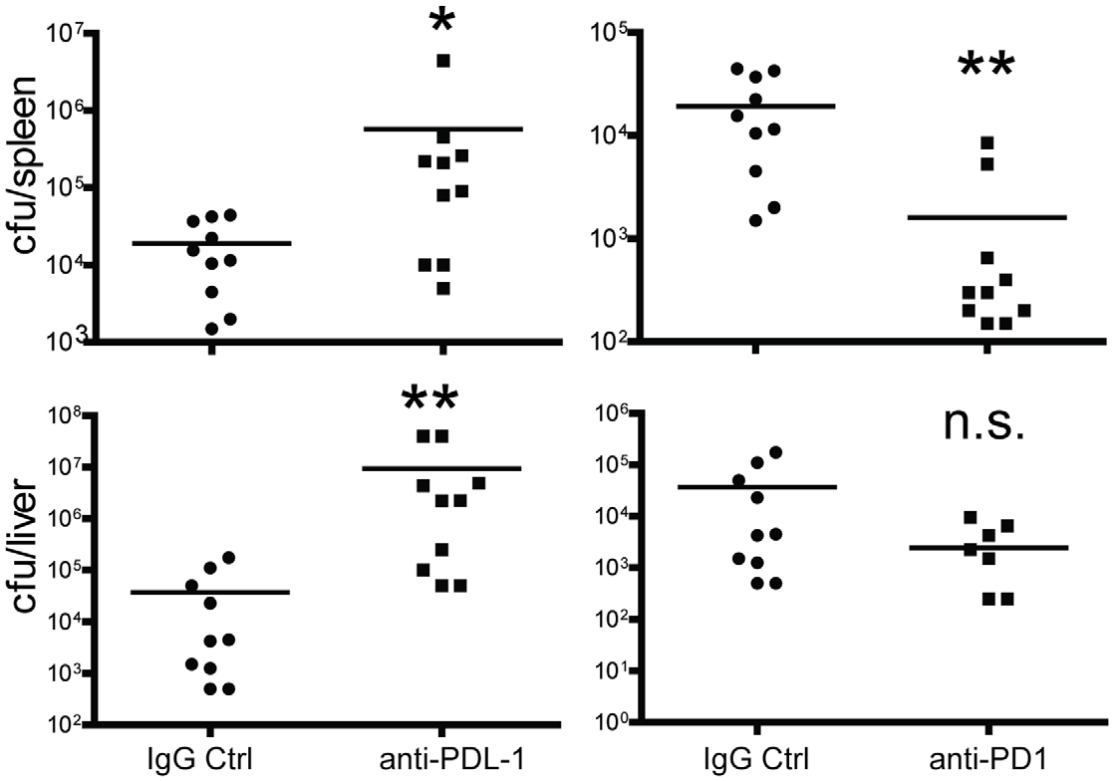
PD-L1 costimulation augments protection against LM infection. Mice were infected with 1×10^5^ cfu LM-OVA i.v. and treated with anti-PD-L1, anti-PD1 or control IgG. The bacterial burden in spleen and liver was analyzed five days later. Data are representative of two independent experiments with ten mice per group. Data were analyzed by Mann-Whitney test. (*p<0.05, **p<0.01, ns, not significant).

Effector T cell heterogeneity is a hallmark of CD8 T cell responses to infections [21]. Based on KLRG1 and IL-7R expression levels, four populations of effector cells can be identified: early effector cells (KLRG1- IL-7R-; EEC) that give rise to the other subsets, short-lived effector cells (KLRG1+ IL-7R-; SLEC) that do not survive long-term, memory precursor effector cells (KLRG1− IL-7R+; MPEC) that survive to form the memory pool, and double positive effector cells (KLRG1+ IL−7R+; DPEC) whose origin is unclear [22]. A number of factors have been identified that affect the lineage decision toward MPEC vs. SLEC development [21], [23]. We therefore examined whether PD-L1 played a role in effector subset development in response to LM infection. Blockade of PD-L1 resulted in a decrease in all effector subsets with the greatest effect on SLEC generation (Fig. 4A,B). Moreover, blockade of PD-L1 during LM infection impaired bacterial clearance, while PD-1 blockade enhanced bacterial clearance in the spleen and liver (Fig. 5). This finding further indicated distinct functions for PD-1 and PD-L1 during the anti-LM response.

**Figure 6:**
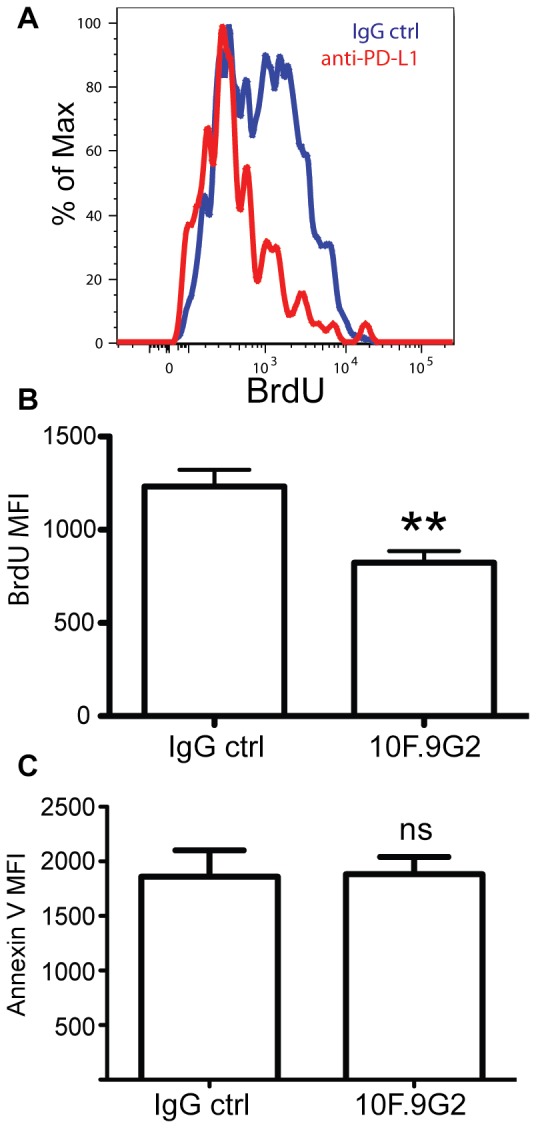
PD-L1 augments Ag-specific CD8 T cell proliferation. A, and B, Brdu incorporation of OVA tetramer+CD8+ T cells. Mice were administered BrdU 16 hrs before sacrificing on day 5 after i.v. LM infection with or without PD-L1 blockade. C. Annexin V staining of tetramer+ cells. Data were analyzed by Student's *t* test, (**p<0.01). Data are representative of three independent experiments with five mice per group.

To further understand the mechanism of PD-L1 costimulation we examined early proliferation of antigen-specific CD8 T cells. To this end, we administered BrdU to infected mice 16hrs before sacrifice with or without PD-L1 blockade. Incorporation of BrdU into CD8 T cells was analyzed on day 5 post-infection(Fig. 6A,B). While most tetramer+ cells from the control mice incorporated BrdU, fewer cells incorporated BrdU after PD-L1 blockade (Fig. 6A). Furthermore, in those Ova/K^b^-specific CD8 T cells that did incorporate BrdU during PD-L1 blockade the level of incorporation was reduced (Fig. 6B). Using annexin V staining, no difference in apoptosis was observed between the groups (Fig. 6C). Thus, PD-L1 costimulation operated via enhancement of proliferative pathways.

### PD-L1 costimulates CD8 T cells independent of CD4 T cell help

**Figure 7:**
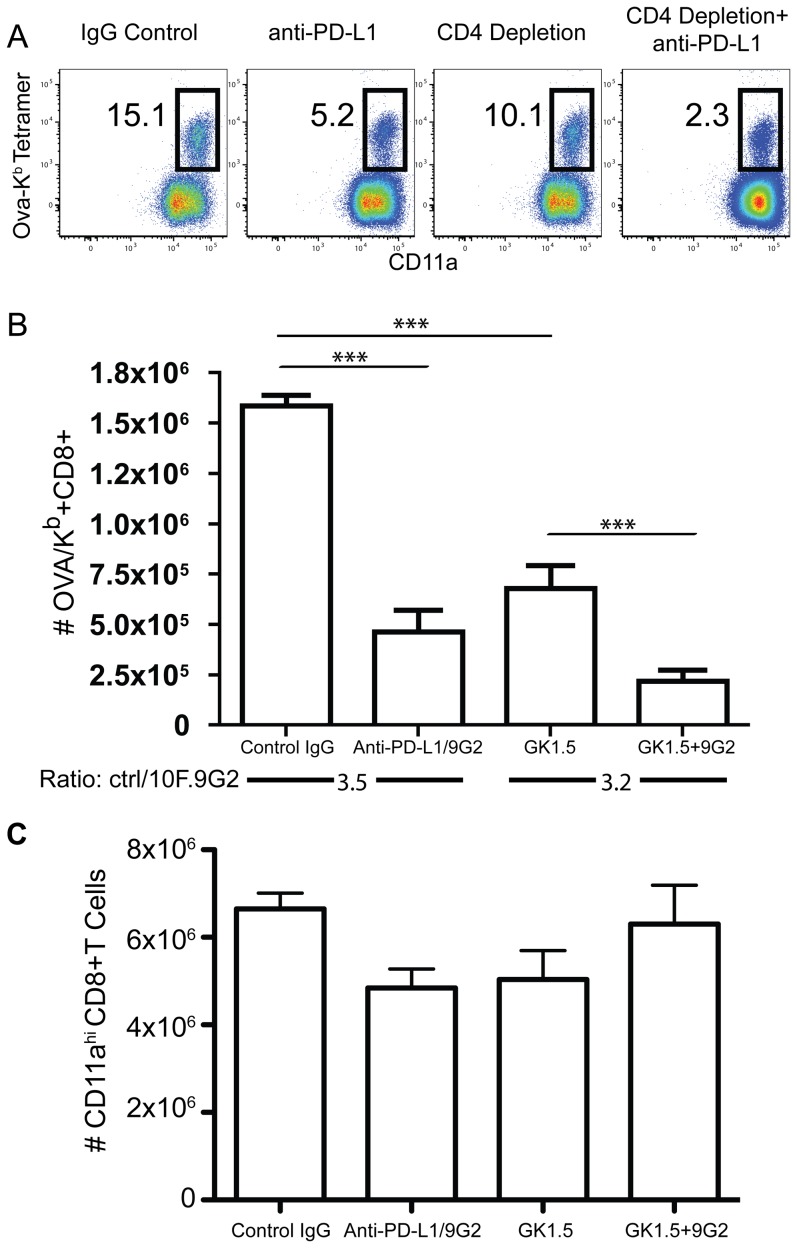
PD-L1 costimulation is independent of CD4 T cell help. A, Representative dot-plots of the antigen-specific CD8 T cell response eight days after LM-OVA infection following PD-L1 blocking with or without CD4 T cell depletion. B, The total numbers of OVA_257–264_/K^b^-specific CD8 T cells or panel C, the total numbers of CD11a^high^ CD8 T cells cells in the spleen from day 8 LM infected mice treated with IgG isotype control, anti-PD-L1 (10F.9G2), anti-CD4 (GK1.5), or both anti-PD-L1 and anti-CD4. Comparison of the magnitude of blocking between PD-L1 blockade with or without CD4 T cell depletion is shown under the bar graph in panel B. Data are representative of three independent experiments with five mice per group. ***p<0.001.

PD-L1 preferentially costimulated the CD8 T cell response with little effect on the CD4 T cell response (Fig. 2). Since the CD8 T cell response to LM is CD4 T cell dependent [24], we next tested whether PD-L1 operated independently or cooperatively with CD4 T cells to augment the CD8 T cell response. To test this, we blocked PD-L1 separately or in conjunction with CD4 T cell depletion. While both treatments inhibited the response, anti-PD-L1 blockade was somewhat more effective than CD4 depletion (Fig. 7A,B). However, CD4 T cell depletion together with anti-PD-L1 blockade substantially enhanced the inhibitory effect of either treatment alone. We further calculated the ratio of antigen-specific CD8 T cell numbers with or without PD-L1 blockade and CD4 T cell depletion. The level of inhibition was similar in the presence or absence of CD4 T cells (Fig. 7B). We noticed that the CD11a expression on tetramer-negative CD8 T cells appeared to increase after PD-L1 blockade or CD4 depletion (Fig. 7A). However, the total number of splenic CD11a^high^ CD8 T cells was not different between the groups (Fig. 7C), suggesting that CD11a upregulation might be non-specific and the result of alterations in the inflammatory environment. Overall, these data indicated that both PD-L1 costimulation and CD4 T cell help were required for optimal CD8 T cell responses to LM infection.

### PD-L1 costimulation occurs independent of binding to known epitopes of PD-1 and CD80

**Figure 8:**
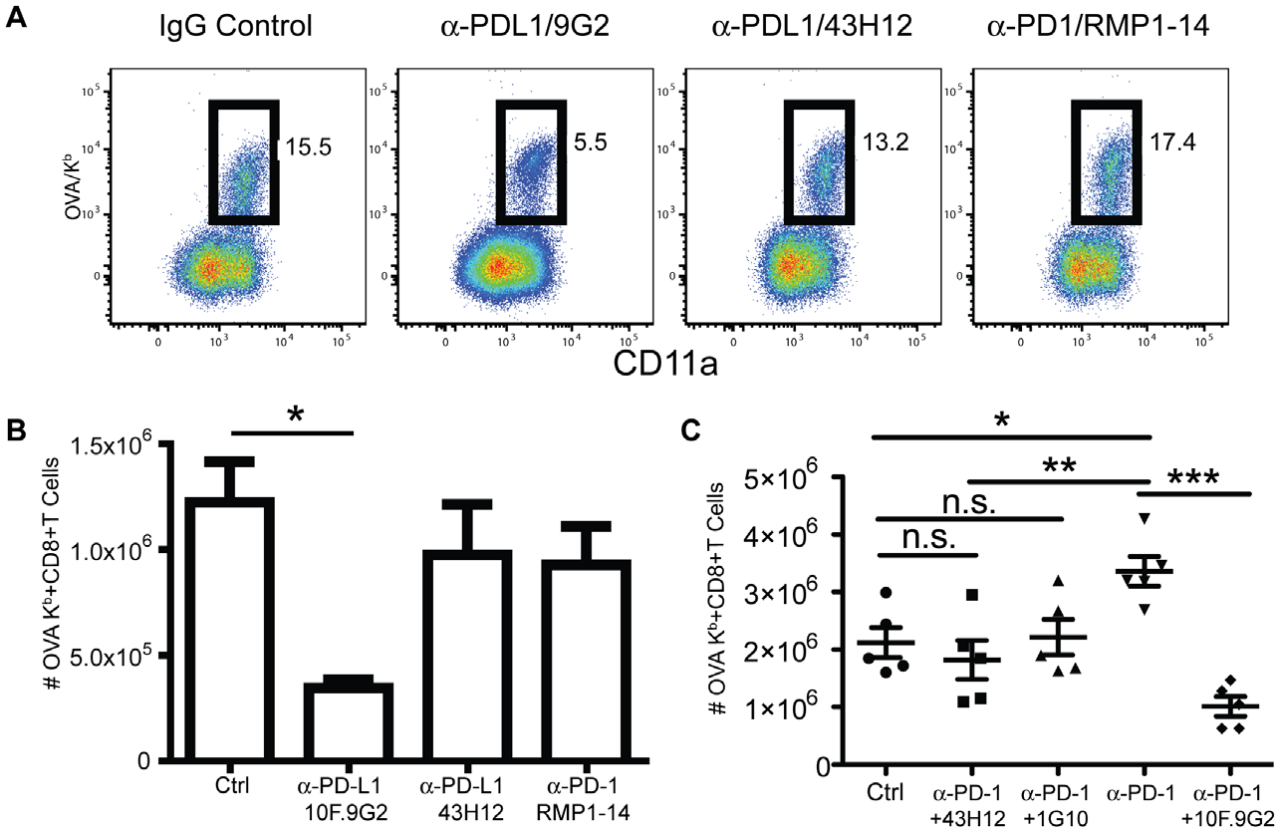
PD-L1-mediated costimulation occurs independently of known CD80 or PD-1 interactions. Representative dot-plots (A) and total cell numbers (B) of the OVA_257–264_/K^b^-specific splenic CD8 T cell response eight days after LM infection from mice treated with IgG isotype control, anti-PD-L1 (10F.9G2), or with an mAb that blocks PD-L1 interaction with CD80 (43H12), or with anti-PD-1 (RMP1-14). C, Total numbers of OVA_257–264_/K^b^-specific splenic CD8 T cells eight days after LM infection from mice treated with IgG isotype control, anti-PD-1 (RMP1-14), anti-PD-L1 (10F.9G2), both anti-PD-L1(10F.9G2) and PD1(RMP1-14), both anti PD-1(RMP1-14) and 43H12 or both anti-PD-1(RMP1-14) and anti-CD80 (1G10). Data are representative of three independent experiments with five mice per group. Data were analyzed by two-way ANOVA, (*p<0.05, **p<0.01, ***p<0.001, n.s., not significant).

The two known counter-receptors of PD-L1 are PD-1 and CD80, both of which are well documented to transduce negative regulatory signals during T cell activation [4], [7]. To scrutinize through which ligand PD-L1 mediated costimulation, we took advantage of mAbs that specifically block PD-L1 binding to PD-1 (RMP1-14; [16]) or to CD80 (43H12); [7]) and compared their ability to block the CD8 T cell response during LM infection with the general inhibition of PD-L1 by 10F.9G2. Surprisingly, treatment with either RMP1-14 or 43H12 failed to inhibit the response unlike 10F.9G2 treatment (Fig. 8A,B,C). As an important positive control, we confirmed the blocking efficiency of 43H12 in a previously described T cell tolerance model [7]. Treatment with 43H12 greatly enhanced the CD8 T cell response in this model (data not shown). In addition, the consistent increase in the CD4 T cell response (data not shown) and enhanced LM clearance (Fig. 5) with RMP1-14 treatment, indicated that this mAb was also operating. To insure that the lack of inhibition of the CD8 T cell response by PD-L1-CD80 blockade (43H12) or PD-1 blockade (RMP1-14) was not due to compensation through CD80 or PD-1, we blocked both interactions simultaneously, and found no inhibition (Fig. 8B). This result was also confirmed by blocking CD80 with 1G10 (Fig. 8B), which has been shown to block CD80:PD-1 interaction in vitro[8]. In this experiment, anti-PD-1 treatment resulted in an increase in antigen-specific CD8 T cells (Fig. 8B), but this was not a consistent finding. Further, to exclude the possibility that the reduced antigen-specific CD8 T cell response was caused by a potentiated inhibitory effect via enhancing PD-L1:PD-1 interaction due to 10F.9G2 mAb treatment, we blocked PD-1 in conjunction with 10F.9G2 treatment which again demonstrated that 10F.9G2 blockade of PD-L1 reduced the antigen-specific CD8 T cell response (Fig. 8B). Taken together, these data suggested that PD-L1 costimulation was mediated either by binding to an epitope on CD80 or PD-1 that was not blocked by the available mAbs or by interaction with a third unknown binding partner.

While much research has focused on the inhibitory effects of the PD-L1/PD-1 axis, positive costimulatory effects of these and other predominantly negative regulators have also been described. The underlying reasons that determine negative versus positive regulatory events are not clear. Our studies show obvious contextual cues that control the requirement for PD-L1 mediated costimulation since the CD8 T cell response to VSV infection was PD-L1 independent while the response to LM infection integrated positive signals from PD-L1 costimulation (Fig. 1). Previous work also showed a role for PD-L1 costimulation in the CD8 T cell response to LM infection[9], [10] that is mediated through IFNγ[11]. Expression of counterligands that direct the choice between positive and negative regulation may be differentially controlled during distinct immune responses. While the identity of the putative third PD-L1 ligand is not yet known, the expression of this counterreceptor could be disparate between, for example, VSV and LM infection resulting in the different outcomes of the CD8 T cell response during PD-L1 blockade that we observed. This ligand may be distinct from PD-L1 and CD80 or could represent an interaction between PD-L1 and epitopes on these molecules that remain accessible in the presence of the available blocking antibodies. This possibility is supported by the finding that PD-1-deficient CD8 T cells also exhibit a defect in expansion in the response to LM infection[11]. Nonetheless, the ultimate effect was enhancement of the response, indicating a distinction in the downstream signaling events mediated through PD-L1 interactions which drive negative regulatory events versus the positive effects described here.

Our data also indicated that both positive and negative regulation were occurring simultaneously through PD-1 and PD-L1. Thus, while PD-L1 inhibition reduced the overall CD8 T cell response and decreased protection, PD-1 blockade enhanced bacterial clearance without consistently affecting the overall magnitude of the CD8 T cell response. The latter result suggested that PD-1 may be inhibiting the functional abilities of CD8 T cells or was affecting innate immune system components. Of additional significance was the demonstration that PD-L1 costimulation operated cooperatively, but independently of CD4 T cell help. Thus, the summation of the positive and negative signaling events mediated through PD-1/PD-L1 family members served to fine-tune the overall immune response to provide protection while maintaining the integrity of the host.
